# RNA Binding Proteins and Regulation of mRNA Translation in Erythropoiesis

**DOI:** 10.3389/fphys.2018.00910

**Published:** 2018-07-24

**Authors:** Kat S. Moore, Marieke von Lindern

**Affiliations:** Department of Hematopoiesis, Sanquin Research, and Landsteiner Laboratory, Amsterdam UMC, Amsterdam, Netherlands

**Keywords:** erythropoiesis, mRNA translation, translation initiation factors, eIF4E, eIF2, CSDE1, polyadenylation

## Abstract

Control of gene expression in erythropoiesis has to respond to signals that may emerge from intracellular processes or environmental factors. Control of mRNA translation allows for relatively rapid modulation of protein synthesis from the existing transcriptome. For instance, the protein synthesis rate needs to be reduced when reactive oxygen species or unfolded proteins accumulate in the cells, but also when iron supply is low or when growth factors are lacking in the environment. In addition, regulation of mRNA translation can be important as an additional layer of control on top of gene transcription, in which RNA binding proteins (RBPs) can modify translation of a set of transcripts to the cell’s actual protein requirement. The 5′ and 3′ untranslated regions of mRNA (5′UTR, 3′UTR) contain binding sites for general and sequence specific translation factors. They also contain secondary structures that may hamper scanning of the 5′UTR by translation complexes or may help to recruit translation factors. In addition, the term 5′UTR is not fully correct because many transcripts contain small open reading frames in their 5′UTR that are translated and contribute to regulation of mRNA translation. It is becoming increasingly clear that the transcriptome only partly predicts the proteome. The aim of this review is (i) to summarize how the availability of general translation initiation factors can selectively regulate transcripts because the 5′UTR contains secondary structures or short translated sequences, (ii) to discuss mechanisms that control the length of the mRNA poly(A) tail in relation to mRNA translation, and (iii) to give examples of sequence specific RBPs and their targets. We focused on transcripts and RBPs required for erythropoiesis. Whereas differentiation of erythroblasts to erythrocytes is orchestrated by erythroid transcription factors, the production of erythrocytes needs to respond to the availability of growth factors and nutrients, particularly the availability of iron.

## Gene Expression and Signal Transduction Set the Stage for Erythropoiesis

Erythrocytes circulate through human peripheral blood for approximately 120 days. Every day, the human body produces 10^11^ new erythrocytes to replenish the cells that are taken out of the circulation by macrophages. This requires a tight balance between progenitor proliferation and maturation, in conjunction with erythropoietin (Epo) dependent cell survival. Erythroid homeostasis is controlled by a cascade of transcription factors and by signaling pathways triggered by environmental factors.

**FIGURE 1:**
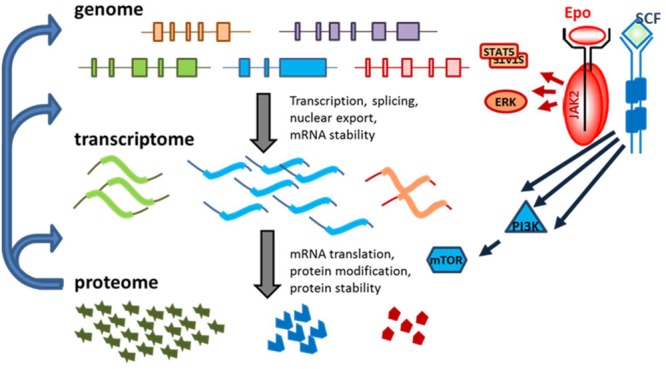
Different levels of regulation of gene expression in erythropoiesis. Cell-specific transcription factors control which genes are expressed. Transcript levels per gene differ because of transcription rate, but also due to mRNA stability. Transcript levels poorly predict protein expression. Translation factors and mRNA structure control protein synthesis efficiency. Transcript and protein expression is partly cell intrinsically regulated as a cascade of subsequent gene expression regulation (left hand arrows). In addition, signal transduction (Epo and SCF in erythroid progenitors) can modify signaling molecules to activate transcription and translation. For instance, phosphorylation of STAT5 causes dimerization and activates the transcription activity, ERK phosphorylation cause nuclear location and phosphorylation of transcription factors. PI3K activates mTOR, a critical factor in protein synthesis.

Several transcription factors that are specifically expressed in erythroid progenitors, are essential for determining the erythroid program of gene expression (**Figure 1**). For instance, GATA-1 (GATA-binding factor 1) functions as one of the master regulators of erythropoiesis, inducing for instance (i) the expression of the Epo receptor (EpoR), (ii) commitment to the erythroid lineage, and (iii) transcription of the β-globin locus (Gregory et al., 1999; Ferreira et al., 2005; Kim and Bresnick, 2007; Ribeil et al., 2013). GATA1 protein synthesis is reduced in Diamond-Blackfan anemia (DBA), a congenital red cell aplasia caused by reduced biosynthesis of ribosomes, and mutations in GATA1 can cause a DBA-like phenotype (Ludwig et al., 2014; Vlachos et al., 2014; Paolini et al., 2016). Also of importance, particularly during terminal erythroid differentiation, is KLF1 (Kruppel-like factor 1, or E-KLF: erythroid KLF), a transcription factor involved in many cellular changes required for the maturation of erythroblasts to erythrocytes, including expression of β-globin (Dzierzak and Philipsen, 2013; Perkins et al., 2016). Haploinsufficiency in KLF1 causes hereditary persistence of fetal hemoglobin (HPFH) (Borg et al., 2010), and a specific mutation in the DNA-binding domain of KLF1 causes type IV congenital dyserythropoietic anemia (CDA) (Iolascon et al., 2013).

Signaling cascades allow for the adaption of the gene expression program laid down by transcription factors such as GATA1 and KLF1 during erythropoietic expansion and differentiation upon demand (**Figure 1**). For instance, during hypoxic stress, Epo production in the kidneys is dramatically increased (Sposi, 2015). The binding of Epo to its cognate receptor (EpoR) activates the cytoplasmic tyrosine kinase JAK2 (Witthuhn et al., 1993), which triggers a downstream signaling cascade via the signal transducer and activator of transcription 5 (STAT5), phosphoinositide-3-kinase (PI3K), and protein kinase B (PKB) pathways to support the proliferation and differentiation of erythropoietic progenitors (Sui et al., 2000; Menon et al., 2006; Fang et al., 2007; Socolovsky et al., 2007). STAT5 induces the expression of, for instance, anti-apoptotic B-cell lymphoma-extra large (BCL-X_L_) to maintain viability during terminal maturation (Gregory et al., 1999). Recently, many erythroid specific STAT5 targets were identified (Gillinder et al., 2017). Stem cell factor (SCF), the ligand for the SCF receptor KIT, acts in conjunction with Epo to repress differentiation and promote expansion of the population of erythroid progenitors (Broudy et al., 1996). SCF signaling is enacted via the PI3K pathway, resulting in the activation of PKB (Sui et al., 2000; van Dijk et al., 2000). PI3K/PKB signaling has two major effector pathways in erythroblasts. On the one hand, it prevents nuclear localization of the Forkhead box O3 (FOXO3) transcription factor that otherwise induces erythroid differentiation (Bakker et al., 2004). Concurrently, the PI3K/PKB signaling pathway activates mammalian target of rapamycin (mTOR), which interacts with translation initiation factors to promote the translation of a specific set of transcripts (Gingras et al., 1999; Grech et al., 2008).

Studies on gene expression have historically focused on transcription as the primary regulatory mechanism. However, it has become increasingly clear that protein levels correlate poorly with transcript expression, and that translation is a major determinant of protein abundance in the cell (Schwanhäusser et al., 2011; Brar and Weissman, 2015). In other words, DNA transcription determines whether a given gene is expressed and determines the fundamentals of the gene expression program. Subsequently, mRNA translation has this program at its disposal for control of protein synthesis. Finally, protein modification and degradation also control protein expression (**Figure 1**). This review focusses on specificity in control of translation, which is generated by the combination of nucleotide sequences in the 5′ and 3′ untranslated regions (5′UTR, 3′UTR), together with expression levels and posttranslational control of constitutive translation factors and RNA binding proteins (RBPs). Control of mRNA translation is mediated by a complex web of overlapping mechanisms, including, but not limited to, regulation of translation initiation and the length of the poly(A) tail, by the presence of short upstream open reading frames (uORFs), and by sequence-specific association with RBPs. In this review, a brief overview of these topics of selective mRNA translation as pertains to erythropoiesis will be presented.

## Translation Initiation Factors in Cap-Dependent Protein Synthesis

**FIGURE 2:**
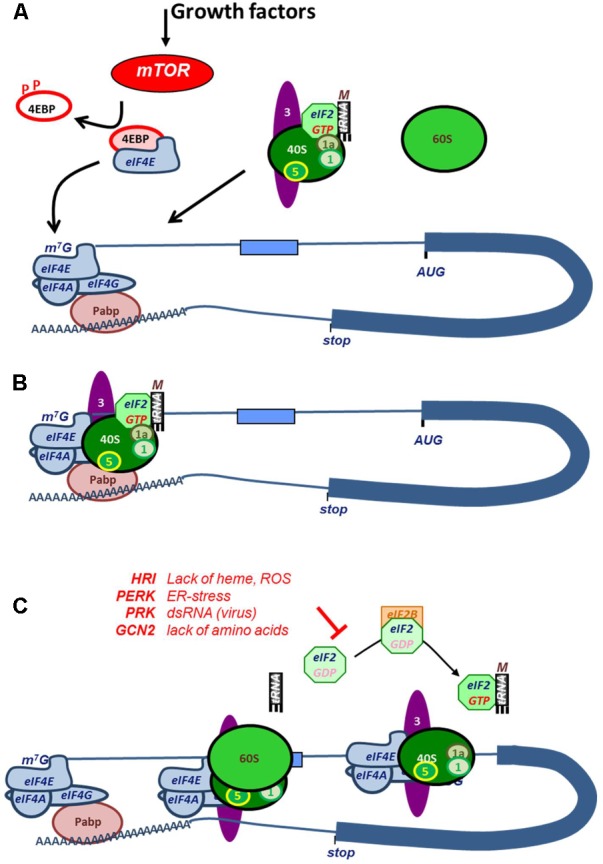
Mechanism and regulation of cap-dependent translation initiation. **(A)** Growth factor signaling activates phosphorylation of 4EBP by mTOR, which releases the cap-binding factor eIF4E. Upon binding to the mRNA cap, eIF4E associates with helicase eIF4A and scaffold protein eIF4G to form the eIF4F complex. The interaction between the poly(A)-tail binding protein PABP with eIF4G forms a closed loop between cap and tail. Concurrently, the ternary complex, existing of eIF2:GTP and tRNA_i_^met^, associates with the 40S ribosomal subunit and eIF1, -1A, -3, and -5 (labeled numerically in the figure). This assembly is referred to as that 43S pre-initiation complex. **(B)** eIF3 interacts with eIF4G in the eIF4F complex. After docking with the ribosome, the 48S pre-initiation scanning complex is formed, and the transcript is scanned until encountering a start codon, upon which eIF2-bound GTP is hydrolyzed and the 60S ribosomal subunit is recruited. At this stage, initiation of translation is complete, and translational elongation begins. **(C)** Cap-dependent translation is regulated by the phosphorylation of eIF2. After a round of translation initiation, eIF2 must be recharged with GTP by eIF2B in order for the ternary complex to be reformed and for the 60S ribosomal subunit to be recruited in subsequent cycles of translational initiation. Under stress conditions, eIF2 is phosphorylated by, for example, HRI (heme regulated inhibitor, during heme scarcity), PKR (Protein kinase R, upon recognition of viral dsRNA), PERK (PRK-like endoplasmic reticulum kinase, upon aggregation of malfolded proteins), or GCN (General control non-derepressible, during amino acid starvation), preventing eIF2B from exchanging eIF2-bound GDP for GTP. The lack of unphosphorylated eIF2 leads to translational arrest.

Canonically, translation is initiated via an interaction between the 5′ 7-methylguanylate (^7m^G) cap structure of the mRNA and the cap-binding protein eukaryotic initiation factor 4E (eIF4E) (Kong and Lasko, 2012). Interaction of eIF4E with the cap is the major rate limiting step, which is followed by binding of scaffolding protein eIF4G, and the RNA helicase eIF4A to form the eIF4F complex. The scaffolding protein eIF4G in this complex interacts with poly-A binding protein (PABP) to form a closed-loop structure between the 5′ and 3′ end of the mRNA (**Figure 2**). Concurrently, the ternary complex (TC), consisting of the methionine loaded initiator tRNA (tRNA_i_^met^) and the GTP-loaded GTPase eIF2 associates with the 40S ribosomal subunit and with translation factors eIF1, -1A, -3, and -5 to form the 43S pre-initiation complex (**Figure 2**; Hinnebusch, 2014). The preinitiation scanning complex, also referred to as the 48S initiation complex, is subsequently formed via an interaction between eIF4G and eIF3. The preinitiation scanning complex scans the bound mRNA until encountering an initiation codon, at which point recognition of the initiation codon triggers the hydrolysis of eIF2-bound GTP by eIF5 and the recruitment of the 60S large ribosomal subunit, marking the end of initiation phase and the beginning of elongation (**Figure 2**).

Next to eIF4E, the availability of GTP-loaded eIF2 is an important rate limiting factor of translation initiation (Proud, 2005). Once translation initiation starts, the TC dissociates upon hydrolysis of GTP to GDP. To reassociate with the TC and rejoin the scanning complex, eIF2 must be reloaded before a new round of translational initiation can begin. This is accomplished via GDP-GTP exchange factor eIF2B, allowing the pre-initiation complex containing eIF2-GTP and tRNA_i_^met^ to reform. This mechanism is subject to control by phosphorylation of the α subunit of eIF2, which prevents the GDP-GTP exchange, resulting in the inhibition of cap-dependent translation (**Figure 2**).

The control of rate-limiting factors eIF4E and eIF2 in erythropoiesis and the corresponding signaling pathways are discussed in the next two paragraphs.

### Epo and SCF Controlled Translational Initiation in Erythroblasts

**FIGURE 3:**
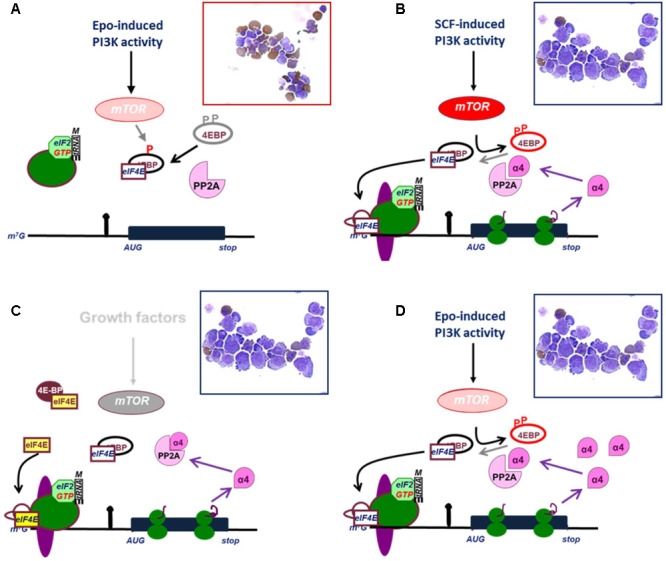
Release of eIF4E and expression of IGBP1/alpha4 are effectors of mTOR activation. **(A)** Epo induces weak PI3K/mTOR signaling which causes only partial phosphorylation of 4EBP and fails to release eIF4E. The protein phosphatase PP2A dephosphorylates 4EBP and thereby inhibits eIF4E. In absence of eIF4E, 43 kD complex (see legend **Figure 2**) cannot associate with mRNA. As a result, erythroid progenitors differentiate (hemoglobin is stained brown in cytospin). **(B)** Epo plus SCF induces full phosphorylation of 4EBP and release ofeIF4E, which enables asembly of the preinitiation scanning complex at the mRNA cap, and translation of *IGBP1* mRNA to produce IGBP1 also known as the alpha4 inhibitory subunit of PP2A. **(C)** Overexpression of eIF4E out-titrates 4EBP and enables translation of *IGBP1* in absence of mTOR activation. **(D)** Constitutive expression of IGBP1/Alpha4 inhibits dephosphorylation of 4EBP and enables weak PI3K/mTOR activation to result in full phosphorylation of 4EBP, release of eIF4E and translation of IGBP1/Alpha4. **(B–D)** IGBP1 is an example representing many other transcripts with highly structured 5′UTRs. Cytospins indicate that translation of IGBP1 is associated with proliferation of erythroid progenitors in absence of differentiation.

Stem cell factor (SCF) cooperates with Epo to expand erythroblast numbers *in vivo* and *in vitro*, whereas erythroblasts mature to erythrocytes in presence of Epo only (Wessely et al., 1999). The crucial pathway activated by SCF is the mTOR-dependent release of the cap binding factor eIF4E from its binding protein 4EBP (Blázquez-Domingo et al., 2005). Overexpression of eIF4E inhibits erythroid differentiation (Blázquez-Domingo et al., 2005; **Figure 3**). This is in part due to SCF-independent proliferation of erythroblasts, but also due to a block in erythroid differentiation (Chung et al., 2015). Upon release from 4EBP, eIF4E binds the mRNA cap-structure, allowing the formation of the pre-initiation scanning complex (Hinnebusch, 2014; Siddiqui and Sonenberg, 2015). In addition to release of eIF4E, PI3K dependent phosphorylation of eIF4B increases its association with the helicase eIF4A and enhances eIF4A activation, which facilitates scanning of the 5′UTR (Shahbazian et al., 2010). Thus, PI3K activation increases formation of the preinitiation complex at the cap, but also increases the capacity of the complex to unwind structures in the 5′UTR. The increased scanning capacity controls overall translation initiation, but transcripts with a TOP (terminal oligopyrimidine tract) or secondary structures are hypersensitive to PI3K activity and eIF4E availability (Graff and Zimmer, 2003; Modelska et al., 2015).

One such transcript is *immunoglobulin binding protein* (*IGBP1*), which is constitutively expressed in erythroblasts, but selectively translated only upon SCF-induced eIF4E release (Grech et al., 2008). IGBP1 is more suitably known as the alpha4 subunit of protein phosphatase 2A (PP2A) (Nanahoshi et al., 1998). As a regulatory subunit, it inhibits the catalytic activity of PP2A on 4EBP (**Figure 3**). Therefore, expression of IGBP1/Alpha4 enhances the effect of low levels mTOR activation on mRNA translation. SCF-induced expression of IGBP1 acts as a positive feedback mechanism in the polysome recruitment of multiple eIF4E-sensitive mRNAs, resulting in SCF-independent proliferation of erythroblasts and attenuation of erythroid differentiation (Grech et al., 2008). Another transcript subject to the mTOR/eIF4E pathway and crucial for erythroid differentiation encodes USE1 (Unusual SNARE protein in the ER 1), which controls retrograde vesicle transport between the Golgi apparatus and the endoplasmic reticulum (Grech et al., 2008; Nieradka et al., 2014). Finally, also mitochondrial biogenesis is under control of the mTOR/eIF4E pathway in erythroblasts (Liu et al., 2017).

### eIF2 Phosphorylation in Erythropoiesis

Regulation of translation initiation is of particular importance in hemoglobin synthesis. Erythrocytes carry approximately 250 million hemoglobin molecules, each consisting of 4 globin peptides and 4 iron-containing heme molecules. Iron deficiency reduces heme availability and presents a risk of cell damage from the accumulation of free α- and β-globins that form toxic precipitates known as Heinz bodies (Jacob and Winterhalter, 1970). Therefore, in- and export of iron in erythroblasts, and the synthesis of heme and globin needs to be tightly coupled (Chen, 2014). Heme acts as a signaling molecule which binds to eIF2 associated kinase (eIF2ak1), also called HRI (Heme Regulated Inhibitor) (Han et al., 2005). During heme deficiency, HRI phosphorylates the α-chain of trimeric eIF2, preventing the exchange of eIF2-bound GDP for GTP by eIF2B and therefore also preventing the reassociation of the TC and the preinitiation scanning complex (**Figure 2**; Chen, 2007; Chung et al., 2012). This results in a global inhibition of protein synthesis (Liu et al., 2008). This mechanism is similar to eIF2 alpha phosphorylation resulting from detection of (viral) double-stranded RNA via eIF2ak2 (PKR), ER-stress via eIF2ak3 (PERK), and amino acid starvation via eIF2ak4 (GCN2) (Wek et al., 2006). Control of eIF2 phosphorylation is crucial during erythroid differentiation. Mice carrying alleles in which control of eIF2 phosphorylation is disturbed suffer from anemia. Phosphorylation of eIF2 induces translation of *ATF4* (Activating transcriptional factor 4), and expression of its target *PPR15a* (*Protein phosphatase 1, regulatory subunit 15A*, also known as *GADD34*). GADD34 enhances the enzyme activity of the catalytic PP1 subunit PPR15b (Harding et al., 2009). Mice that lack ATF4 suffer from mild compensated anemia, whereas mice lacking HRI or GADD34 are anemic with inefficient erythropoiesis (Masuoka, 2002; Patterson et al., 2006; Zhang et al., 2018). Mice lacking the PP1 catalytic subunit die perinatally with severe anemia (Harding et al., 2009).

### Upstream Open Reading Frames

Increased sensitivity to translation factors, and particularly to eIF2, is predicted by the presence of uORFs in the 5′UTR of the transcript. The uORFs render mRNA translation hypersensitive to translation initiation factors at the level of (i) start codon recognition, (ii) interaction of the peptide or the uORF sequence with the ribosome, and (iii) reinitiation of the TC (eIF2:GTP and tRNA_i_^met^) with the scanning complex (Hinnebusch, 2014; Young et al., 2015). Translation of the uORF first requires recognition of the start codon. The better a start codon is embedded in a Kozak consensus sequence, the longer the scanning complex pauses, and the higher the chance that the GTPase activity of eIF2 is activated and translation is initiated (Kozak, 1986). Not only the local nucleotide sequence, also a downstream secondary structure may delay the scanning while the eIF4A helicase activity unfolds the transient obstacle. Finally, the activity of the eIF2 GTPase activity is controlled by eIF1 and eIF5 that regulate eIF2 activity and specificity. Interestingly, expression of eIF1 and -5 is controlled by SCF and during erythroid differentiation (Grech et al., 2008). Translation of the uORF causes eIF2 to dissociate from the complex, and requires reassociation of a new TC with the scanning complex before the scanning complex reaches the start codon of the next ORF. Fast reassociation depends on eIF2 expression and phosphorylation (**Figure 2C**). In cases where the uORFs overlaps with the protein-coding ORF (indicated as CDS, coding sequence), start codon recognition is mostly in a less favorable Kozak consensus sequence, whereas the presence of multiple uORFs in the 5′UTR typically renders translation dependent on the distance between uORFs and between the uORF and the CDS.

Whereas translation is generally inhibited upon eIF2 phosphorylation, the translation of specific transcripts may be enhanced under these conditions. One such transcript is *ATF4*, which is essential for erythroid differentiation and for reduction of oxidative stress during the basophilic erythroblast stage (Suragani et al., 2012). ATF4 contains two uORFs, the second of which overlaps with the CDS (Vattem and Wek, 2004). Translation of the second uORF inhibits ATF4 protein expression by overwriting the CDS (Lu et al., 2004). Phosphorylation of eIF2 decreases translation of the second uORF and increases translation of the *ATF4* CDS ORF. Whether uORFs are translated, has to be determined experimentally. Ribosome footprint analysis is a novel breakthrough in this field, because it provides the position of ribosomes on a transcript, and can identify which uORFs are actually translated (Ingolia, 2014). Treatment of erythroblasts with tunicamycin to induce eIF2 phosphorylation via PERK reduces the overall density of ribosomes on the transcriptome (Paolini et al., 2018). The ribosome density increases, however, on at least 140 transcripts among which *ATF4*, but also *PNRC2* (*proline rich nuclear receptor coactivator 2*, involved in RNA stability) and *IFDR1* (*interferon-related developmental regulator 1*, involved in cellular stress resistance) (Paolini et al., 2018). Other uORF-containing transcripts, instead, are hypersensitive to eIF2 phosphorylation and translation decreases more than average upon eIF2 phosphorylation in erythroblasts, among which *CSDE1* (*Cold shock domain protein e1*, see below), *PABPC1* (*PolyA binding protein c1*), and *PAPOLA*, [*poly(A) polymerase alpha*], which suggests that eIF2 phosphorylation may result in shortening of mRNA polyA-tails. The response to eIF2 phosphorylation is cell type specific, as indicated by a comparison between ribosome footprints in erythroblasts and HEK293 cells (Andreev et al., 2015; Paolini et al., 2018).

The analysis of uORF translation in erythroblasts indicates that uORFs are involved in eIF2-dependent translational regulation, but they do not predict eIF2-dependent translational regulation. This characterizes the advances of the field. We increasingly understand how general translation factors act together to control cap-dependent translation in commonly used cell models. For specific transcripts we understand the interplay of general translation initiation factors and specific sequences or structural elements in the 5′UTR of the transcript. However, when this information is used to make general predictions, the predictions are often wrong. For instance, Mfold is a well-known program for making structural predictions (Zuker, 2003). This is a useful tool, but chaperones and RBPs present in specific cell types may be more important determinants of structures and the energy required to unfold these structures. Therefore, we need to increase our understanding on the role of sequences and structures in the 5′UTR that have often been neglected when promoter studies were performed to understand gene expression and only the protein coding ORF of a transcript was used to study the role of the encoded protein.

### Alternative 5′UTR Through Erythroid Specific Promoters or Alternative Splicing

The use of erythroid specific promoters and alternative splicing of the 5′UTR is common and has largely been ignored when it does not affect protein identity. It has been estimated that up to one third of all genes expressed in erythroid progenitors may be expressed from an alternative promoter (Tan et al., 2006; Cheng et al., 2014). The presence of an alternative 5′UTR can include or exclude structural elements (stem-loop structures or uORFs), with major consequence for translation initiation. RBP involved in alternative splicing of the 5′UTR are also modifiers of translation. Notably, erythroid differentiation is associated with extensive alternative splicing (Pimentel et al., 2014), with a prominent role for the RBP Muscleblind (Cheng et al., 2014). Alternative splicing mainly involves coding sequences (Cheng et al., 2014), but may specifically alter translation regulation in the 5′UTR. For instance, translation and stability of *Heme Oxygenase-1* (*HO1*) is regulated by hemin as part of feedback control. Alternative splicing generates a hemin resistant alternative transcript (Kramer et al., 2013). Similarly, transcript variants of *Ferroportin* are expressed during erythropoiesis that vary in their 5′UTR to enable iron responsive and non-responsive translation (Cianetti et al., 2005). In case of the SNARE-complex protein USE1, a predicted G-quadruplex in an alternatively spliced 5′UTR results in USE1 protein expression mainly from the small ratio of transcripts with intron retention (Nieradka et al., 2014). Alternative splicing can also alter the translation initiation codon that is used. Detection of alternative translation start sites can be established by ribosome footprinting or by mass spectrometry, in which case novel predicted N-termini must first be added to the reference library (Van Damme et al., 2014; Floor and Doudna, 2016).

## Ires Trans-Activating Factors

In addition to cap-dependent translation, ribosomes can associate on a subset of transcripts that carries an internal ribosomal entry site (IRES) (Komar and Hatzoglou, 2011). While there is no consensus sequence or universal structural motifs for IRESs, they typically contain complex structural elements which include stem loops and pseudoknots (Komar and Hatzoglou, 2005; Baird et al., 2007). The majority of IRESs are found within the 5′UTR directly upstream of the initiation codon, though they can also exist within the coding region, causing synthesis of a truncated protein (Komar et al., 2003; Grover et al., 2009). IRES-mediated translation is preferred under conditions of decreased availability of cap-dependent translation (Lewis and Holcik, 2008; Spriggs et al., 2008), due, for example, to eIF2 phosphorylation (Gerlitz et al., 2002; Tinton et al., 2005) or cleavage of scaffolding protein eIF4G (Komar and Hatzoglou, 2005). This includes, but is not limited to, viral infection, hypoxia, nutrient starvation, ER stress, and cell differentiation (Komar and Hatzoglou, 2005; Graber and Holcik, 2007).

Translation via an IRES requires the binding of IRES *trans*-activating factors (ITAFs) such as Polypyrimidine Tract Binding protein (PTB) (Bushell et al., 2006; Cobbold et al., 2010) and CSDE1 (Hunt et al., 1999; Mitchell et al., 2003; Cornelis et al., 2005). Some ITAFs are required to unfold the secondary structure, which subsequently enables other ITAFs to bind to specific sequence elements, and to recruit the ribosomal subunits. IRES-mediated translation initiation is less competitive in ribosome recruitment. Compared to the ^7m^G cap structure, IRES elements are less competitive to recruit ribosomes. It is probably for that reason that primarily IRES-dependent translation initiation is suppressed when less ribosomes are present, which is a hallmark of DBA (Horos et al., 2012). The induction of severe anemia due to loss of ribosomes indicates that IRES-mediated translation is of particular importance in erythropoiesis.

Several genes involved in hematopoiesis are subject to IRES-mediated translation. BCL2-associated athanogen 1 (BAG1) and Heat Shock Protein 70 (HSP70) cochaperone are required for terminal differentiation of erythroblasts (Horos et al., 2012). All three BAG1 isoforms are produced from a single transcript dependent upon the involvement of either cap-dependent or IRES-mediated translation (Coldwell et al., 2001). Two ITAFs are involved in this process: PCBP1 [poly(rC) binding protein 1], which remodels the RNA to allow ribosomal entry, and PTB, which is necessary for the recruitment of the ribosome to the *BAG1* transcript (Pickering et al., 2004). Bag1 deficiency is lethal at day E13.5 in mice because of a complete lack of definitive erythropoiesis (Götz et al., 2005). shRNA-mediated knockdown of BAG1 in erythroblasts results in the production of fewer hemoglobinated daughter cells under differentiation conditions.

Another example of IRES-mediated translation is transcription factor RUNX1, which is essential for fetal liver hematopoiesis (Okuda et al., 1996). The *RUNX1* gene has two promoters that yield RUNX1 transcripts with two distinct 5′UTRs, one of which contains an IRES (Pozner et al., 2000; Levanon and Groner, 2004). The absence of the IRES in Runx1 causes embryonic fatality due to disordered proliferation and differentiation of hematopoietic cells in the fetal liver (Nagamachi et al., 2010).

Internal ribosomal entry sites have a unique role in circular RNAs that are generated by backsplicing. Without head and tail, these circular RNAs are hardly degraded, and lack the potential of being translated by cap-dependent mRNA translation. Interestingly, some circular RNAs encode proteins because translation initiation of circular RNA can be initiated from an IRES (Legnini et al., 2017; Pamudurti et al., 2017; Begum et al., 2018).

## RBPs Control mRNA Stability and Translation Through the mRNA Poly(A) Tail

**FIGURE 4:**
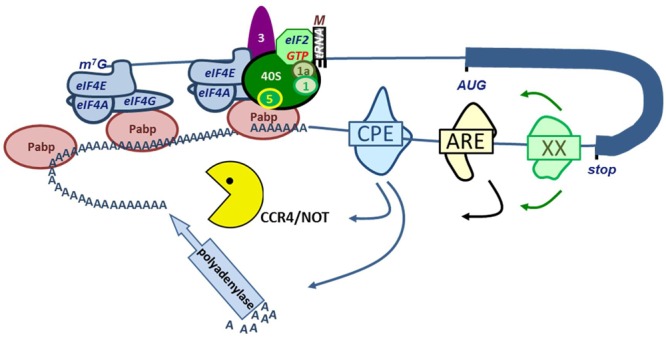
RNA binding proteins (RBP) control mRNA stability and translation. Interaction of eIF4G in the eIF4F complex with PABP results in a closed loop model of mRNA translation (see legend **Figure 2**). Binding of PABPs to the poly(A) tail enhances mRNA stability and translation. A variety of RBP binding to e.g., the cytoplasmic polyadenylation element (CPE), AU-rich Elements (ARE) or other element represented as XX. These RPB interact with polyadenylases or with the CCR4/NOT deadenylating complex.

Almost all protein coding mRNAs (except histones) are polyadenylated at their 3′ end. The poly(A) tail protects from degradation, and the length of the poly(A) tail also affects translation initiation (**Figure 2**). The length of the poly(A) tail is determined by the recruitment of polyadenylating and deadenylating enzymes to the transcript. Polyadenylation initially occurs in the nucleus where mRNA cleavage and polyadenylation are coupled (Charlesworth et al., 2013). Upon cleavage site recognition, a nuclear poly(A) polymerase is recruited to synthesize the poly(A) tail. Typically this is carried out by PAPOLA, but depending on the cell type and specific transcript, another, non-canonical polymerase may be involved (Charlesworth et al., 2013). Upon export to the cytoplasm, RBP binding to the 3′UTR may recruit polyadenylating or deadenylating proteins that may enhance or repress translation of the mRNA transcript. A broad array of RNA-binding proteins governs this process. Here, an overview of some of the larger families of poly(A)-interacting proteins and their influence on erythropoiesis will be presented (**Figure 4**).

### PABPs Promote Translational Initiation and Stabilize Transcripts via poly(A) Binding

Essential to the initiation of translation is the binding of poly(A) binding proteins (PABPs) to the poly(A) tail. PABPs directly interact with the eIF4G scaffold protein of the eIF4F cap-binding complex (Hinnebusch and Lorsch, 2012). This brings the mRNA tail close to the cap, and forms a mRNA loop conformation that is believed to optimize recycling of translation initiation and elongating factors (Wakiyama et al., 2000). The interaction of PABP with eIF4G stabilizes pre-initiation scanning complexes on the 5′UTR (Kahvejian et al., 2005; **Figure 2**). A longer poly(A) tail increases PABP affinity for target transcripts, which enhances the stabilization of multiple preinitiation scanning complexes and thereby enhances translation initiation efficiency. Additionally, the binding of PABP to the poly(A) tail protects the transcript from deadenylation and degradation (Norbury, 2013), a process which is discussed in more detail below.

The most common PABP is PABPC1. In erythroid MEL (mouse erythroid leukemia) cells, however, there is a prominent role for Pabpc4 (Kini et al., 2014). Pabpc4 binds to a specific subset of transcripts with short poly(A) tails containing an AU-rich motif, including α-globin, and protects them from further degradation. Depletion of Pabpc4 blocks induced terminal differentiation of MEL cells by altering the expression of five genes associated with erythroid maturation. Receptor tyrosine kinase c-Kit is strongly upregulated in Pabpc4-depleted MEL cells. This activity is specific to Pabpc4 and not redundant with Pabpc1. Given that downregulation of c-Kit is essential for terminal differentiation (Munugalavadla et al., 2005; Dzierzak and Philipsen, 2013), the rescue of c-Kit expression is likely responsible for the inability of Pabpc4-depleted MEL cells to mature (Kini et al., 2014). Also induced were c-Myb, c-Myc, CD44, and Stat5a, all well-studied genes which promote erythroblast maintenance at the expense of differentiation (Hattangadi et al., 2011).

### CPEBs Control poly(A) Tail Length

Among the proteins able to recruit cytoplasmic polyadenylation enzymes are the cytoplasmic polyadenylation element binding proteins (CPEBs). CPEBs, however, are promiscuous proteins able to recruit either cytoplasmic poly(A) polymerases such as the Drosophila GLD2 protein (Germ Line Development 2; TENT2, terminal nucleotidyltransferase 2, in man; PAPD4, PAP associated domain containing 4 in mouse), which promotes mRNA stability and translation, or the deadenylating CCR4/NOT complex (Barnard et al., 2004; Ogami et al., 2014; **Figure 4**). Whether binding of CPEBs increases the length of the poly(A) tail or causes deadenylation depends on the interaction with additional RBP such as PUMILIO, or on phosphorylation of CPEB, for instance by Aurora kinase A (AURA) (Charlesworth et al., 2013). Deadenylation by CPEB depends on recruitment of the CCR4/NOT complex that is composed of several CNOT subunits, which individually have varying roles in a myriad of physiological processes. CAF1 and CCR4 are subunits with deadenylation activity (Shirai et al., 2014). In particular, CNOT9 was identified as an erythropoietin-responsive gene, indicating a role for the complex in erythropoiesis (Gregory et al., 2000). Deadenylation initially represses translation, because less PABP can bind and connect to eIF4G proteins in preinitiation complexes. Ultimately, deadenylation results in silencing of gene expression via mRNA degradation (Shirai et al., 2014).

Of the CPEB family members, CPEB4 is specifically induced during erythroid differentiation (Bakker et al., 2007; Hu et al., 2014). Erythroid expression of CPEB4 is regulated via the transcription factors GATA1 and TAL1 (Hu et al., 2014), its strong upregulation late in differentiation depends on FOXO3 (Bakker et al., 2007; Kerenyi and Orkin, 2010; Hattangadi et al., 2011). Because the recruitment of polyadenylating or deadenylating complexes depends on the context of the transcripts, i.e., on the complexes formed with RBP that bind to nearby elements in the 3′UTR, CPEB4 enhances or decreases mRNA translation with transcript-specific regulation. In erythroblasts, CPEB4 also associates with the eIF3 complex and the major mechanism appears to be repression of translation via the interaction with eIF3 (Hu et al., 2014). Both over- and underexpression of CPEB4 impairs the terminal differentiation of erythroblasts to reticulocytes, indicating that the level of CPEB4 needs to be controlled. Interestingly, CPEB4 is capable of binding to and repressing its own mRNA, forming a feedback loop that maintains CPEB4 levels within a range required for terminal erythropoiesis (Hu et al., 2014).

Although CPEBs are generally considered as factors that recruit adenylating or deadenylating enzymes, interaction with other general translation factors may also contribute to regulation of translation. Whereas CPEB4 binds eIF3, CPEB1 is known to increase mRNA stability by binding to PABPC1 and PABPC1L (Seli et al., 2005; Guzeloglu-Kayisli et al., 2008).

### Musashi-Mediated Translational Control in Hematopoiesis

In addition to CPEB and PABP, cytoplasmic polyadenylation is regulated by MUSASHI-1 and -2 (MSI1 and MSI2). These proteins do not specifically control erythropoiesis, but they are very important for hematopoietic stem- and progenitor cells (HSPC) from which erythropoiesis develops. MSI1 and MSI2 interact with mRNA via the MSI-binding element (MBE) (Charlesworth et al., 2013). The MBE element is known to confer cytoplasmic polyadenylation in the absence of CPEB activity (Charlesworth et al., 2006). The poly(A) polymerase GLD2 interacts with MSI in Xenopus oocytes, but interactions between MSI proteins and the polyadenylation machinery have been described in mammalian cells (Charlesworth et al., 2013; Cragle and MacNicol, 2014).

The majority of studies done on MUSASHI-mediated translational repression have been done with MSI1. MSI1 represses the translation of target transcripts by competing with eIF4G for binding with PABP, preventing the formation of the 80S ribosome subunit (Kerwitz et al., 2003). Transcripts silenced by Msi1 include cell cycle regulators such as Numb, an inhibitor of the NOTCH pathway, and the cell cycle inhibitor p21 (Bardwell et al., 1990; Topalian et al., 2001). RNA binding domains between MSI1 and MSI2 are largely homologous (85–95%), but MSI2 has no PABP-binding domain (de Andrés-Aguayo et al., 2012). However, there is evidence to suggest that MSI2 alters NOTCH localization and upregulates HES1, a NOTCH reporter protein (Kharas et al., 2010), suggesting that MSI1 and MSI2 overlap in regulating common targets.

MSI2 is abundantly expressed in primitive LSK cells of the hematopoietic lineage, where MSI1 expression is nearly absent (Kharas et al., 2010; de Andrés-Aguayo et al., 2011; Hope and Sauvageau, 2011). The expression of MSI2 is subsequently downregulated during differentiation. Downregulation of MSI2 alters the balance between self-renewal and differentiation of HSCs via regulation of the NOTCH pathway (Kharas et al., 2010; Hope and Sauvageau, 2011). This effect is achieved without influencing apoptotic rates or homing behavior. A mouse line expressing a truncated MSI2 gene (*Msi2^Gt/Gt^*) results in a marked decrease in short term hematopoietic stem cells (ST-HSCs) and multipotent progenitors (MPPs) while the effect on long term hematopoietic stem cells (LT-HSCs) was nominal (de Andrés-Aguayo et al., 2011). MSI2-defective LSKs display impaired proliferation, and the LT-HSC population is decreased following non-competitive bone marrow transplantation (Kharas et al., 2010). In addition, a doxycycline-inducible MSI2 transgenic mouse model observed an increase in ST-HSC/MPP populations and a decrease in LT-HSC, whereas MSI2 overexpression increased LT-HSC self-renewal in transplanted mice. Taken together, these findings suggest that MSI2 is important for HSPC self-renewal and stem cell homeostasis particularly during stress hematopoiesis. Studies on MSI2 in hematopoiesis have been largely functional in focus and do not mechanistically investigate mRNA polyadenylation via MSI2. Although the direct molecular targets of MSI2 in hematopoiesis are unknown, gene expression profiling indicates a regulatory function for pathways involved in HSC proliferation, including MEIS1, HOXA9, HOXA10 (Hope and Sauvageau, 2011), RAS, MAPK, CyclinD1, and MYC (Kharas et al., 2010; de Andrés-Aguayo et al., 2011). The role of MSI2 in Hematopoiesis has been extensively reviewed in de Andrés-Aguayo et al. (2012).

### AUBPs in Hematopoiesis

The AAU1/HNRPD family of AU-rich element binding proteins (AUBPs) are oppositely regulated from the MSI proteins (Sakakibara et al., 2002). AU-rich elements (AREs) are sequence motifs (typically AUUUA) in the 3′UTR that recruit a large family of AU-binding proteins (AUBPs) that regulate translation (**Figure 4**). They were among the first sequence elements known to affect stability of mRNA (Shaw and Kamen, 1986). Translational regulation via AUBPs can occur via a number of mechanisms including deadenylation, and transcript sequestration to P-bodies and stress granules (Baou et al., 2011). AUBPs key to erythropoiesis include the tristetraprolin (TTP) family members (ZFP36, ZFP36L1, and ZFP36L2) and HuR/ELAV1.

TTP family members interact with NOT1 to promote the rapid deadenylation by the CCR4/NOT complex (Sandler et al., 2011; Fabian et al., 2013). Interestingly, ZFP36L1 and ZFP36L2 demonstrate opposite regulation in erythropoiesis. ZFP36L2 is expressed in erythroid progenitors in response to glucocorticoids and downregulated during terminal differentiation. ZFPL1, instead, is only expressed in more mature erythroblasts (Zhang et al., 2013; MvL unpublished data). Concordantly, ZFP36L2 is required for burst-forming unit-erythrocyte (BFU-E) renewal (Zhang et al., 2013), whereas ZFP36L1 downregulates STAT5B expression, reducing the formation of erythroid colonies (Vignudelli et al., 2010). Why these highly homologous factors have such opposite effects in erythropoiesis, and how they regulate the transcripts to which they are bound, is unclear. In keratinocytes, ZFP36L1 and ZFP36L2 also have non-redundant roles in the regulation of growth factor expression, but in T cells, both ZFP36L1 and ZFP36L2 inhibit cell proliferation by inhibiting the expression of cell cycle genes, particularly D Cyclins (Galloway et al., 2016; Prenzler et al., 2016).

ELAV1, also known as HuR, is a ubiquitously expressed AUBP with thousands of direct and functional targets (Lebedeva et al., 2011; Mukherjee et al., 2011). Relevant transcripts bound by ELAV1 encode proteins that control cell cycle progression, apoptosis, signal transduction, and lineage specific transcription factors (reviewed in Baou et al., 2011). Interestingly, ZFP36L1 is a functional target of ELAV1 in cells exposed to oxidative stress, wherein ionizing radiation decreases *ZFP36L1* transcript binding by ELAV1, resulting in a decreased recruitment of *ZFP36L1* to polysomes (Mazan-Mamczarz et al., 2011). This suggests that ZFP36L1 may synergize with ZFP36L1 in regulating erythropoiesis under some conditions.

### Alternative Polyadenylation Controls mRNA Stability and Translation

The examples mentioned above are only a brief and incomplete overview of how proteins binding to the 3′UTR may control mRNA stability and translation through recruitment of polyadenylases or the deadenylating complex. It is long known that the nuclear cleavage and polyadenylation complex may recognize alternative sites for mRNA cleavage resulting in alternative length of the 3′UTR. The exclusion of binding sites for RBPs can have major consequences for mRNA stability and translation (Mayr, 2016). The implications of single transcripts have been studied, but systematic analysis of alternative polyadenylation (APA) and their consequences for mRNA stability are scarce (Sun et al., 2012). Novel methods are available to determine APA sites, but it is difficult to combine for instance ribosome footprint analysis with APA because the output of ribosome footprinting exists of short ribosome protected fragments that cannot be attributes to distinct transcripts. To date only few examples of APA are known in erythropoiesis but nothing is known about the relation of APA and control of gene expression by RBPs (Kudo et al., 1994; Boehmelt et al., 1998).

## RBPs Involved in Additional Control of Translation

In addition to general translation initiation factors and RBP that control the poly(A), mRNAs contain secondary structures and specific sequences that bind RBP to specifically control their translation. An important posttranscriptional mechanism of gene expression is mediated by microRNAs. Argonaut proteins are the miRNA-binding RBPs that associate with RNA. Mice deficient in Argonaut 2 (Ago2) suffer from severe anemia, which is caused by the failure of miR-144/451 to act on its targets (Rasmussen et al., 2010). However, Ago2 is the Argonaut protein with slicing activity and appears to control mRNA stability more than translation (Jee et al., 2018). We therefore focus our discussion on the role of a few selected RBP that are important for erythropoiesis, rather than on miRs.

### Translational Control of Iron Homeostasis

Iron homeostasis in erythroid cells is achieved via a balanced regulation of iron import via transferrin receptor 1 (TfR1), storage in ferritin and export via ferroportin (Kühn et al., 2015). Posttranscriptional control over these proteins is enacted by iron regulatory proteins (IRPs). IRPs are recruited to *ferritin* mRNA via a conserved sequence that forms a hairpin structure in the 5′UTR (Aziz and Munro, 1986; Hentze et al., 1987). Binding of the IRP to the IRE (Iron Response Element) prevents the association of the 43S preinitiation complex to the mRNA transcript (Gray and Hentze, 1994). The presence of iron blocks the IRE-IRP interaction, allowing translation of the formerly repressed ferritin transcript. A similar mechanism governs the translation of *ferroportin* (Abboud and Haile, 2000). Interestingly, a splice variant of *ferroportin* lacking the IRE is expressed in duodenum and erythroid cells permits the escape of IRP-mediated translational control (Cianetti et al., 2005). By contrast, the TfR1 transcript possesses 5 IREs in the 3′UTR rather than the 5′UTR (Koeller et al., 1989; Müllner et al., 1989). Binding of IRPs to *TfR1* confers increased mRNA stability to the transcript, resulting in an inverse relationship between TfR1 protein expression and iron abundancy (Owen and Kühn, 1987; Müllner and Kühn, 1988).

Both IRPs cooperate to control iron homeostasis, yet they are regulated via different mechanisms. IRP1 is a bifunctional protein with both enzymatic and RNA-binding activity. Its possesses the capacity to act as an aconitase in the catalyzation of citrate to isocitrate (Kennedy et al., 1983). The catalytic activity is dependent upon the assimilation of an additional iron atom in the active site, with the result that IRP1 functions as an enzyme when iron is abundant, and as an RNA-binding protein when iron is depleted (Haile et al., 1992; Emery-Goodman et al., 1993; Hirling et al., 1994). Because the formation of the iron-sulfur cluster in the active site requires an oxygen-free environment (Beinert and Kennedy, 1993), the RNA-binding form is preferentially induced in the presence of the vasodilating agent nitrous oxide (Drapier et al., 1993).

Although IRP2 is 57% homologous with IRP1 in humans, it does not function as an aconitase under iron-rich conditions (Guo et al., 1994). Unlike IRP1, IRP2 is rapidly degraded by the proteasome when iron and oxygen levels are high (Guo et al., 1994, 1995). Degradation of IRP2 is prevented by low oxygen pressure (Hanson et al., 2003). Taken together, IRP1 and IRP2 are capable of controlling iron homeostasis under both low and high iron and oxygen supply, allowing a proportional response to environmental stimuli (Kühn et al., 2015).

### The RBP Csde1 Is Essential for Erythropoiesis

CSDE1, originally called UNR (upstream of N-ras) is an RNA-binding protein with five cold-shock domains (Triqueneaux et al., 1999). CSDE1/UNR was initially shown to silence *MSL* (*Male Sex Lethal)* during sex determination of *Drosophila melanogaster* (Jacquemin-Sablon et al., 1994; Gebauer et al., 1999; Grskovic et al., 2003; Abaza et al., 2006; Duncan et al., 2006; Abaza and Gebauer, 2008). Repression of *MSL2* occurs via a direct interaction between CSDE1 and 3′UTR-bound PABP, resulting in the prevention of PABP-mediated recruitment of the 43S complex (Duncan et al., 2009). Paradoxically, the CSDE1-PABP complex has also been reported to stimulate translation by stabilizing the interaction between PABP and eIF4G (Ray and Anderson, 2016).

CSDE1 is implicated in both endogenous and viral IRES-mediated translation in mammalian cells. Both rhinovirus and poliovirus contain IRES elements that require an interaction between CSDE1 and PTB (Hunt et al., 1999; Boussadia et al., 2003). Early studies indicated that all five of CSDE1’s cold-shock domains were necessary for maintenance of CSDE1’s affinity for rhinovirus IRESs, whereas cold-shock domains 1 and 2 had the most impact on CSDE1’s binding to *MSL-2* (Brown and Jackson, 2004; Abaza and Gebauer, 2008), and cold-shock domains 2 and 4 were the only required elements for stimulation of translation via PABP (Ray and Anderson, 2016).

CSDE1 and PTB also cooperate in IRES binding of the *CSDE1* transcript itself, resulting in repression of translation of *CSDE1* (Cornelis et al., 2005; Dormoy-Raclet et al., 2005; Schepens et al., 2007). IRES-mediated translation is promoted during mitosis and apoptosis at the expense of cap-dependent translation (Komar and Hatzoglou, 2005; Tinton et al., 2005). The CSDE1 IRES activity is strongly upregulated during mitosis due to increased binding of hnRNPC1/C2 proteins with simultaneous release of CSDE1 and PTB (Schepens et al., 2007). The resultant increase in Csde1 expression during the G2/M phase of the cell cycle facilitates the IRES-mediated translation of cyclin-dependent PITSLRE kinases, which are essential for centrosome maturation and mitotic spindle formation (Tinton et al., 2005; Petretti et al., 2006). During apoptosis, CSDE1 and PTB upregulate APAF1 (apoptotic protease-activating factor 1) (Mitchell et al., 2001). Binding of CSDE1 and PTB changes the conformation of the APAF1 IRES, granting access for ribosomal recruitment and thereby permitting the translation of the transcript (Mitchell et al., 2003).

CSDE1 is also implicated as a regulator of mRNA stability via poly(A) deadenylation. CSDE1 binds to *cFOS* in conjunction with PABP (Chang et al., 2004). In contrast to the stabilizing influence of PABP when interacting with CPEB, the CSDE1-PABP complex promotes transcript degradation via recruitment of the deadenylase CCR4. Given that binding of PABP and CSDE1 to the poly(A) tail can also increase transcript stability, Chang et al. propose a model in which the CSDE1-PABP complex initially protects the poly(A) tail from deadenylation by CCR4 prior to initiation of translation. Upon ribosomal transit of the mCRD, a conformational change is triggered that forms a landing pad for CCR4, resulting in transcript repression by deadenylation.

CSDE1 also interacts with the 4E-Transporter (4E-T), itself a translational regulator with a wide breadth of functions. 4E-T competitively binds to cap-binding protein eIF4E, preventing its association with scaffolding protein eIF4G (Sonenberg and Hinnebusch, 2009; Kamenska et al., 2016), while simultaneously reducing ribosomal access to the 5′ cap via interaction with RNA-binding proteins at the 3′UTR (Rhoads, 2009; Kamenska et al., 2014b). Furthermore, 4E-T is a component of the CPEB translation repressor complex (Minshall et al., 2007), the CCR4/NOT complex (Kamenska et al., 2014a), and enhances decay of transcripts containing AREs (Ferraiuolo et al., 2005; Chang et al., 2014; Nishimura et al., 2015). 4E-T is directly bound by CSDE1 (Kamenska et al., 2016). 4E-T binding to CSDE1 and CNOT4 is mutually exclusive, which predicts that Csde1 abrogates 4E-Ts role as a bridge between CNOT1 and CNOT4 complex subunits (Kamenska et al., 2016). Due to the complexity of the overlapping pathways associated with 4E-T, it is unclear precisely how CSDE1 cooperates with 4E-T in the regulation of translation. The authors suggest that CSDE1 and DDX6, an RNA helicase component of the CPEB repressor complex, simultaneously bind 4E-T to either redundantly repress translation or to selectively affect specific translational stages. Another possibility is that CSDE1 acts as a competitive inhibitor of 4E-T binding to other, unknown cofactors/repressors, disruption of which unravels a network of interactions necessary for translational repression.

Notably, CSDE1 expression is decreased in erythroblasts with a haploinsufficiency in ribosomal proteins. Expression of CSDE1 is >100-fold upregulated in erythroblasts compared to early progenitors, and expression of Csde1 is required for erythroblast proliferation and differentiation in mouse erythroblasts (Horos et al., 2012). Csde1-bound transcripts and associated proteins that are identified using Csde1 pulldown, encode proteins involved in ribogenesis, mRNA translation and protein degradation, but also proteins associated with the mitochondrial respiratory chain and mitosis (Moore et al., 2018). RNA expression and/or protein expression of Csde1-bound transcripts are deregulated in MEL clones in which the first cold-shock domain of Csde1 is deleted by Crispr/Cas9. For instance, protein expression of Pabpc1 was enhanced while *Pabpc1* mRNA expression was reduced indicating more efficient translation of *Pabpc1* followed by negative feedback on mRNA stability (Moore et al., 2018). Under the conditions used for Csde1 pull down experiments, Csde1 associates most prominently with Strap (serine threonine kinase receptor associated protein). Strap does not affect Csde1/mRNA association, but it alters expression of some transcripts and/or proteins (Moore et al., 2017). In addition to Strap, also Pabpc1 and Pabpc4 associate with Csde1 in MEL cells.

### Heterogeneous Nuclear Ribonucleoproteins hnRNPK and GRSF1

Among the first RBP that were identified and characterized are members of the large superfamily of heterogeneous nuclear ribonucleoproteins (hnRNPs). Within this superfamily four different RNA binding domains and several auxiliary domains are discerned, on basis of which 16 independent families are identified by capital letters (Geuens et al., 2016). The hnRNP family members do not specifically control translation. They are involved in many levels of gene expression regulation including gene transcription, mRNA splicing, mRNA nuclear-cytoplasmic transport, mRNA stability, and translation.

The RBPs hnRNPE1 and hnRNPK are important for late erythroid maturation. Together they control translation of the reticulocyte enzyme 15-lipoxygenase (r15-LOX) (Ostareck et al., 2001). This enzyme is at the base of mitochondrial degradation through dioxygenation of phospholipids in mitochondrial membranes (Grüllich et al., 2001). The silencing of *r15-LOX* translation by hnRNPK involves association of *r15-LOX* with DDX6 in P-bodies, and is regulated by SRC kinase through an intricate feed-back control of hnRNPK and SRC (Naarmann et al., 2008). Translational regulation of mitochondrial degradation in late erythroblasts and reticulocytes is not limited to control of *r15-LOX* translation. Expression of Non-muscle myosin heavy chain IIA (NmhcIIA), involved in intracellular vesicle transport, is also regulated by hnRNPK (Naarmann-de Vries et al., 2016). Thus, the maturation of reticulocytes depends on hnRNPE1 and hnRNPK. These are the only two subtypes of the hnRNP superfamily that carry the unique RNA binding domain indicated as a K-homology domain (KH) (Geuens et al., 2016).

The subfamilies hnRNPF and hnRNPH share a high degree of homology and contain 3 QRRM domains (Quasi RNA recognition motif) (Geuens et al., 2016). Interestingly, hnRNPF/H members were prominently detected among G-quadruplex binding proteins (von Hacht et al., 2014). Similar to hnRNP proteins, the G-quadruplex structure that can occur in DNA and RNA is widely employed to control gene expression, mRNA splicing, transport, stability, and translation (Song et al., 2016). In translational control, they are mainly detected in transcripts expressed during mitosis, and particularly in transcripts that are well expressed in tumor cells (Wolfe et al., 2014). The Guanine-rich RNA sequence binding factor 1 (GRSF1) contains three RNA binding domains and is a member of the heterogeneous nuclear ribonucleoprotein F/H family (hnRNPF/H) (Ufer, 2012; Sofi et al., 2018). GRSF1 binds a G-rich element in *Glutathione Peroxidase 4* (*GPX4*), and in the transcript encoding *Unusual SNARE protein in the ER-1* (*USE1*) (Ufer et al., 2008; Nieradka et al., 2014). Translation of *USE1* depends on the PI3K/mTOR pathway, and on the availability of eIF4E in erythroblasts. Constitutive expression of a truncated USE1 isoform inhibits erythroid differentiation, whereas clones expressing full length USE1 could not be established (Grech et al., 2008). This suggests that regulation of USE1 expression during erythropoiesis is critical. Differential splicing creates transcripts with a long and short 5′UTR. Although most transcripts are spliced and contain a short 5′UTR, the unspliced *USE1* transcript is predominantly present in polyribosomes (Nieradka et al., 2014). This is due to the presence of a G-rich element that scores high in G-quadruplex prediction models. Both USE1 and GRSF1 are expressed in proliferating erythroblasts and are downregulated during differentiation. Suppression of USE1 and GRSF1 expression in erythroblasts decreases the capacity to undergo renewal divisions, and induces terminal erythroid differentiation instead (Nieradka et al., 2014). Thus, the RBP GRSF1 is crucial to erythropoiesis, either through regulation of *USE1* translation or through regulation of other G-Quadruplex containing transcripts.

### More RBPs and Methods to Decipher Translation Efficiency

Transcripts that are subject to control of translation can be identified by density centrifugation to separate subpolysomal from polyribosomal transcripts. In erythroblasts, this resulted in the identification of numerous transcripts that are dependent on for instance growth factors Epo and SCF for their translation (Joosten et al., 2004; Grech et al., 2008). Detailed mRNA translation, at the nucleotide level, can be determined using ribosome footprinting technology (Ingolia et al., 2011). This technology uncovered changes in mRNA translation efficiency in response to tunicamycin-induced phosphorylation of eIF2 (paragraphs 2.2 and 2.3; Paolini et al., 2018). Ribosome footprinting during terminal differentiation of erythroblasts to reticulocytes revealed several classes of transcripts subject to regulated translation (Alvarez-Dominguez et al., 2017). RNA binding motif 38 (RBM38) is an RBP that is prominently expressed in erythroblast. Its expression steadily increases from basophilic erythroblasts to the reticulocyte stage. Interestingly, a polymorphism in RBM38 is associated with erythrocyte volume (van der Harst et al., 2012). RBM38 is known to be associated with erythroid differentiation through control of mRNA splicing (Heinicke et al., 2013; Ulirsch et al., 2016). Of interest, Rbm38 serves as a tumor suppressor gene, and mice that lack Rbm38 are prone to cancer development. In addition, however, they suffer from anemia (Zhang et al., 2014). RBM38 binds to a UGUGU element in the 3′UTR of its target transcripts and interacts with the scaffold protein eIF4G in the eIF4F cap-binding complex. Therefore, Alvarez-Dominguez et al. (2017) hypothesize that RBM38 interferes with the closed loop model of mRNA translation and thereby decreases the efficiency of translation.

Finally, ribosome profiling also showed that ribosomes fail to be recovered in reticulocytes and therefore accumulate in the 3′UTR (Mills et al., 2016). This is due to loss of ABCE1. This loss of ribosome recovery may be the start of total RNA degradation which characterizes the maturation of reticulocytes to erythrocytes during the first 2 days after release of reticulocytes in the peripheral circulation.

## Conclusion

This review presents a general overview of distinct mechanisms and major players that control mRNA translation in erythropoiesis. However, it is clear that there is still much we have yet to understand regarding mechanisms in mRNA translation. The advent of novel molecular and biochemical technologies has revolutionized the large-scale identification of RBPs and their transcripts. Technologies such as iCLIP (Individual-nucleotide resolution UV crosslinking and immunoprecipitation) and ribosome profiling have the exciting potential to rapidly advance the field.

After transcription, translation functions as an additional regulatory layer of gene expression. Precise translational control enables coordinated protein synthesis in response to environmental signals, or the differentiation stage of cells. It also enables the dissociation of gene transcription and protein synthesis in time, which is of particular importance once gene transcription has ceased in late erythroblasts and reticulocytes undergoing terminal erythropoiesis. We expect that the coming years will provide exciting new insights in selective translation of mRNA during erythropoiesis.

## Author Contributions

All authors listed have made a substantial, direct and intellectual contribution to the work, and approved it for publication.

## Conflict of Interest Statement

The authors declare that the research was conducted in the absence of any commercial or financial relationships that could be construed as a potential conflict of interest.
